# Comparative Transcriptomic Analysis of Developing Cotton Cotyledons and Embryo Axis

**DOI:** 10.1371/journal.pone.0071756

**Published:** 2013-08-20

**Authors:** Xiaoming Jiao, Xiaochun Zhao, Xue-Rong Zhou, Allan G. Green, Yunliu Fan, Lei Wang, Surinder P. Singh, Qing Liu

**Affiliations:** 1 Commonwealth Scientific and Industrial Research Organisation Plant Industry, Canberra, Australia; 2 Biotechnology Research Institute/The National Key Facility for Crop Gene Resources and Genetic Improvement, Chinese Academy of Agricultural Sciences, Beijing, China; Wuhan University, China

## Abstract

**Background:**

As a by product of higher value cotton fibre, cotton seed has been increasingly recognised to have excellent potential as a source of additional food, feed, biofuel stock and even a renewable platform for the production of many diverse biological molecules for agriculture and industrial enterprises. The large size difference between cotyledon and embryo axis that make up a cotton seed results in the under-representation of embryo axis gene transcript levels in whole seed embryo samples. Therefore, the determination of gene transcript levels in the cotyledons and embryo axes separately should lead to a better understanding of metabolism in these two developmentally diverse tissues.

**Results:**

A comparative study of transcriptome changes between cotton developing cotyledon and embryo axis has been carried out. 17,384 unigenes (20.74% of all the unigenes) were differentially expressed in the two adjacent embryo tissues, and among them, 7,727 unigenes (44.45%) were down-regulated and 9,657 unigenes (55.55%) were up-regulated in cotyledon.

**Conclusions:**

Our study has provided a comprehensive dataset that documents the dynamics of the transcriptome at the mid-maturity of cotton seed development and in discrete seed tissues, including embryo axis and cotyledon tissues. The results showed that cotton seed is subject to many transcriptome variations in these two tissue types and the differential gene expression between cotton embryo axis and cotyledon uncovered in our study should provide an important starting point for understanding how gene activity is coordinated during seed development to make a seed. Further, the identification of genes involved in rapid metabolite accumulation stage of seed development will extend our understanding of the complex molecular and cellular events in these developmental processes and provide a foundation for future studies on the metabolism, embryo differentiation of cotton and other dicot oilseed crops.

## Introduction

Cotton is a typical bi-functional economic crop. Although approximately 85% of its farmgate value is derived from its fibre production, cotton is currently the fifth largest oilseed crop and the second most important potential source of plant proteins in the world because of the large production of cotton seed [1]. In recent years, cotton seed has been increasingly recognised to have excellent potential as a source of additional food, feed, biofuel stock and even a renewable platform for the production of many diverse biological molecules for agriculture and industrial enterprises. Typically containing 21% oil and 23% protein, cotton seed is evaluated as a wholesome, nutritious and versatile ingredient in animal feed as well as human food products [2], [3]. Cotton seed oil is widely used as cooking oil and an ingredient in marinades, dressings, pastries, margarines, and shortenings. Whole cotton seed following ginning and the cotton seed meal derived from the oil extraction are widely used as protein source for domestic animals, and in particular, the whole cotton seed has been regarded by the dairy industry as a special feed ingredient with advantageous energy and dietary fibre properties required by the high-producing dairy cow. Therefore, value added cotton seed with broader applications through genetic improvement of both seed production and quality without compromising fibre production is clearly advantageous.

Although histological, morphological, molecular and biochemical studies have provided descriptive information on embryogenesis and seed metabolism in cotton, the molecular and physiological events leading to the seed formation and storage compound accumulation are still far from being completely understood. An in-depth understanding of metabolic events that determine the overall components of the storage reserves in cotton seed is therefore of vital importance for improving the yield, quality and ultimately the value of seed constituents and opens the possibility for significant value-adding by engineering novel attributes into the seed.

The cotton embryo itself is made up of two distinct tissues, the cotyledons and the embryo axis. The cotyledons differentiate into nutrient storage organs and the embryo axis into a miniature plant with a shoot and root meristem that progresses into quiescence as the seed mature. The large size difference between cotyledon and embryo axis that make up the embryo results in the under-representation of embryo axis metabolite and transcript levels in whole embryo samples. Therefore, the determination of gene transcript levels in the cotyledon and embryo axis separately should lead to a better understanding of metabolism in these developmentally diverse tissues. Since the small seed size of model dicot plants such as Arabidopsis and *Medicago truncatula* generally precludes studies of this nature, the transcriptome analysis during cotton seed development can serve as a general dicot model for further understanding of the coordinated and differential gene expression in seed development and metabolite accumulation.

High quality RNA-seq data allows identification and accurate quantification of transcription in developing seeds in numerous plant species, including Arabidopsis [4], soybean [5], Jatropha [6], bitter melon [7], sea buckthorn [8] and castor bean [9]. In the current study we have used Illumina’s deep sequencing to study the comparative gene expression in the developing cotyledon and embryo axis. We anticipate that the comparison of gene expression may also provide a useful resource for identifying and characterising genes that play critical roles during cotton embryogenesis, seed development and metabolite accumulation.

## Results and Discussion

### Reads Generation and Assembly

The developing cotton embryo at 30 days after pollination (DAP) was chosen for transcriptomic analysis, representing the active stage of embryo development and metabolism in cotton. The embryo axis and two cotyledons of a developing embryo were separated manually and total RNAs were isolated and subjected to high-throughput RNA-seq analysis to investigate their transcriptomes. After trimming off the adaptor sequences and removing all the low quality reads with unknown nucleotides larger than 5%, the initial run of Illumina’s deep sequencing generated 53,645,968 and 54,517,790 clean reads from cotton embryo axis and cotyledon, respectively. These reads were 75 bp in mean length. An overview of the sequencing and assembly was shown in the Table 1.

**Table 1:** The summary of sequencing and assembling results.

	Length (bp)	Sample	Q sequence (n)	Total bases(bp)	Average length(bp)	Gap distribution(N/size)
Reads	= 75	embryo axis	53,645,968	4,828,137,120	75	–
		cotyledon	54,517,790	4,906,601,100	75	–
Contig	≥100	embryo axis	194,430	38468642	198	–
		cotyledon	194166	46566204	240	–
Scaffold	≥200	embryo axis	78,596	34306080	436	0.0004–0.4234
		cotyledon	76138	35104451	461	0.0002–0.4166
Unigene	≥300	embryo axis	54142	37414396	691	0.0003–0.2964
		cotyledon	53787	37228130	692	0.0003–0.2964

**Figure 1:**
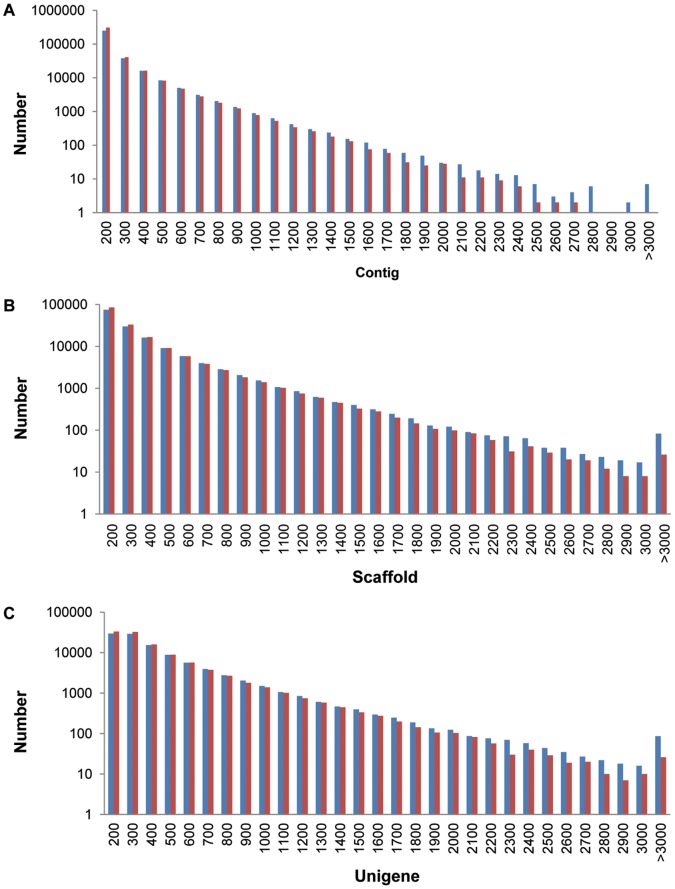
Length distribution of assembled sequences. A, Length distribution of contigs; B, Length distribution of scaffolds; C, Length distribution of unigenes. Samples were derived from: cotyledon (blue), and embryo axis (red).

All the clean reads (108,163,758) were assembled using SOAPdenovo and 388,596 contigs (length >100 bp) were obtained ranging from 100 bp to 4,139 bp in size, with the average length exceeding 198 bp. The assembled reads of both \embryo axis and cotyledon accounted for 45.24% (average length 198 bp) and 54.76% (average length 240 bp) of the corresponding clean reads, respectively. The length distribution of contigs is shown in Figure 1A. Deep sequencing data files are stored in the Sequence Read Archive (SRA) under Study Accession No. SRP026000 that contains sample accession numbers to fastq data files.

A total of 313,899 scaffolds were further assembled using the pair-end information of the assembled contigs. The total number of scaffolds >200 bp in length generated in the embryo axis and cotyledon was 78,596 (average length 436 bp) and 76,138 (average length 461 bp), respectively. Because the scaffolds were produced from contigs using pair-end alignment, it was easier to estimate their length. However, the disadvantage was that some scaffolds sequences contained different percentage of gaps that ranged between 0.02% and 42.34%. In the embryo axis and cotyledon libraries, 29,215 and 28,631 sequences were derived from the high quality assembled scaffolds, respectively. The length distribution of scaffolds is shown in Figure 1B.

The scaffolds were further assembled into unigenes with pair-end annotation. A total 83,831 unigenes more than 300 bp in length, including 54,142 from the embryo axis (average length 691 bp) and 53,787 from cotyledon (average length 692 bp), were used for further analysis. There are 2,240 and 2,234 gapped sequences ranging from min 0.03% to max 29.64%, in the embryo axis and cotyledon, respectively. The length distribution of unigenes is shown in Figure 1C.

**Figure 2:**
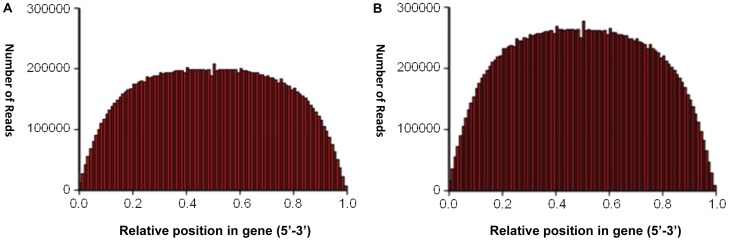
Random distributions of reads on unigenes. The analysis of random distribution of reads on unigenes from: A, cotyledon and B, embryo axis, respectively.

The average sequencing depth calculated by realigning all the sequencing reads to 83,831 assembled unigenes was about 30 folds for each sample. The quality of these unigenes was evaluated by the two following analyses. First, the analysis of random distribution of reads on unigenes from cotyledon and embryo axis indicated an even coverage with evidently fewer reads in the 3′ and 5′ ends (Figures 2A and 2B). Second, the gap distribution analysis indicated that the ratio of gap length to gene length was less than 5% in more than 99% of the unigenes in both the cotyledon and embryo axis libraries.

### Gene Annotation and Function Classification

Using BLASTX program, a set of 83,831 unigenes was searched against all the public protein databases with the e-value cut-off of 0.00001 to find their annotation homologues. A total of 56,439 (67.32%), 35,909 (42.83%) and 15,401 (18.37%) unigenes were aligned against the NCBI Nr, SWISS-PROT, and COG databases, respectively. In both the cotyledon and embryo axis libraries, about one third of all the assembled unigenes had no match in any of the databases (named as “no hit”). 78.84% of these “no hit” sequences were small fragments less than 400 nt in length, some of which possibly derived from the 3′ or 5′ untranslated region of transcripts or non-coding functional RNA. By Blastn search against the miRNA database (http://www.mirbase.org), some known cotton miRNA pre-mature sequences were identified (Table S1). 86% of unigenes in the length range of 500–1,000 nt and 98% of unigenes longer than 1,000 nt, were identified with homologs in the NCBI Nr, SWISS-PROT, and COG databases.

**Figure 3:**
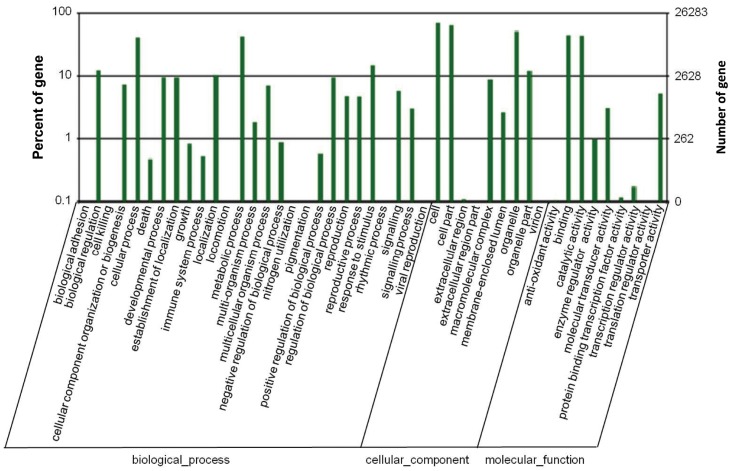
GO function analysis of unigenes from cotton embryo axis and cotyledon. GO terms include “biological process”, “cellular component” and “molecular function”, of which, each was made up of different number of subclasses. The value from the right Y axis of ordinates shows the number of unigenes were classified into each subclass, and the percentage of the number of unigenes from each subclass in the total quantity of unigenes classified into the corresponding term was indicated by the value from the left Y axis of ordinates.

Following the NCBI Nr, SWISS-PROT, and COG annotations, all the unigenes were further checked in the records of the GO (Gene Ontology) database and 24,941 unigenes were retrieved with GO functional annotation. These unigenes were assigned GO terms from the three main ontologies, including 15,907 unigenes with terms from “biological process”, 18,702 unigenes with terms from “cellular component”, and 17,546 unigenes with terms from “molecular function” (Figure 3). Among them, 10,061 unigenes had an assignment in all the three classes. The remaining unigenes were not classified with a GO term, largely because of their uncertain descriptions, such as “unknown”, “putative”, or “hypothetical” protein. Within the “biological process” class, the two most abundant sub-classes were “cellular processes” and “metabolic processes”. Six other subclasses, including “biological regulation”, “developmental processes”, “establishment of localisation”, “locomotion”, “regulation of biological process”, and “response to stimulus” were also enriched with a large number of unigenes. In the “cellular component” ontology, most of unigenes were sorted into “cell part” and “organelle”. In contrast, relatively fewer unigenes were located into “extracellular part” and “virion” subclasses. In the “molecular function” ontology, there are two predominant sub-categories which are represented by genes for “binding” (nucleotide and protein binding) and “catalytic activity” (hydrolase, transferase and kinase activities, etc).

**Figure 4:**
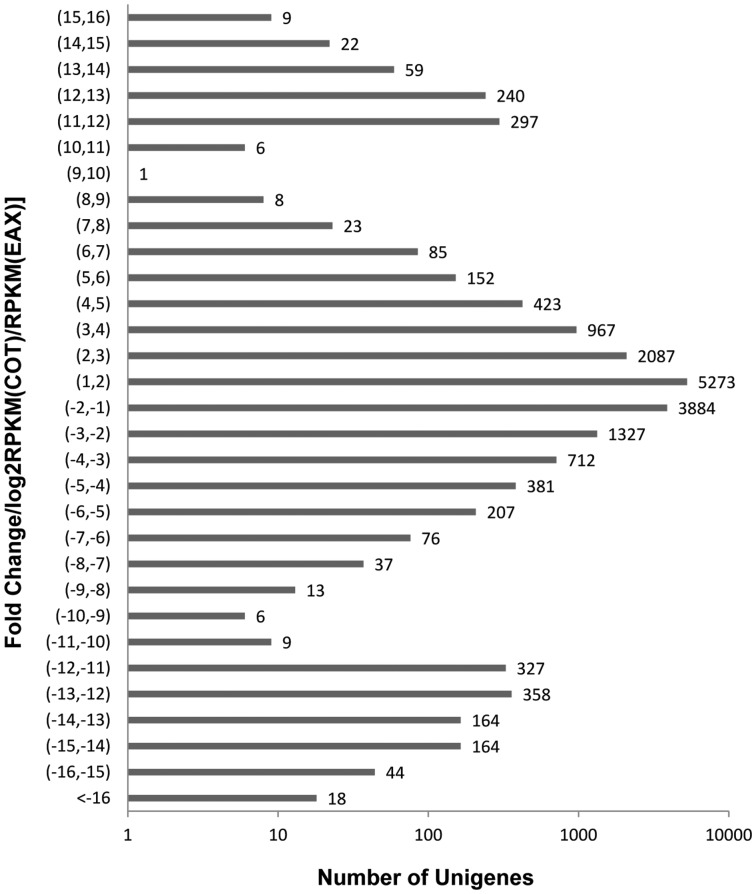
Unigenes that were differentially expressed between cotyledon and embryo axis libraries. Embryo axis abbreviated as EAX, Cotyledon abbreviated as COT, Fold change/EAX/COT (RPKMs) was calculated by the formula: log (RPKM (EAX)/RPKM (COT), 2).

To characterise the differentially expressed unigenes in the transcriptomes of cotton developing embryo axis and cotyledon, the number of reads for each unigene was converted into a normalised RPKM (reads per kilobasepair of transcript per million reads) value, indicating the relative expression level of transcripts in both the tissues studied. We compared the two libraries with the following criteria: absolute value of log_2_ratio >1.0 and P value <0.001. The expression difference between the embryo axis and cotyledon libraries demonstrated that 17,384 unigenes (20.74% of all the unigenes) were differentially expressed in the two adjacent embryo tissues, and among them, 7,727 unigenes (44.45%) were down-regulated and 9,657 unigenes (55.55%) were up-regulated in cotyledon (Figure 4).

### Real time qRT-PCR Analysis for Randomly Selected Unigenes

**Figure 5:**
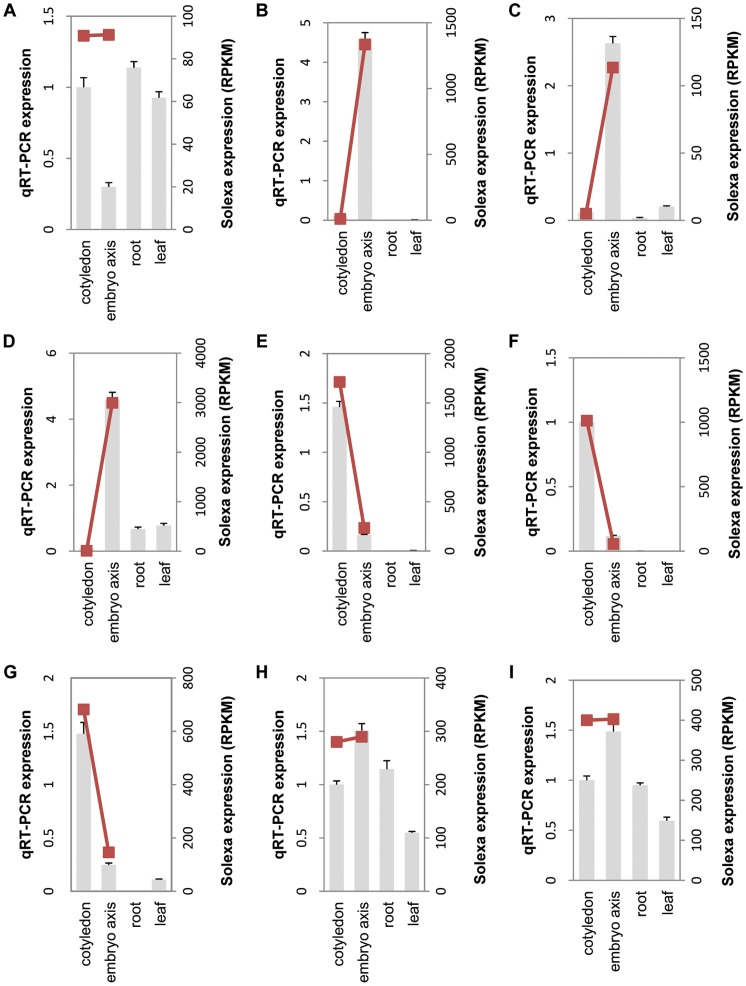
qRT-PCR validations of the selected unigenes. 9 unigenes were selected for qRT-PCR to validate the expression patterns in different samples. The grey bar represent the relative intensity of real time qRT-PCR from independent biological replicates (using the left Y axis), the red squares represent the expression level (RPKM) of the transcript (using the right Y axis) and are connected by the red trend lines. The unigenes are: A, unigene42855; B, unigene70917; C, unigene36175; D, unigene 72799; E, unigene4514; F, unigene37407; G, unigene25168; H, unigene29210 and I, unigene19199.

To validate the RNA-seq results, 30 unigenes were randomly selected for real time quantitative RT-PCR (qRT-PCR) assays from four tissue types, including cotyledon, embryo axis, leaf and root. All the selected 30 unigenes had different expression levels between cotyledon and embryo axis as indicated by the RNA-seq results. As shown in Figure 5, with the exception of unigene42855 (Figure 5A), the comparative expression patterns of the other 29 unigenes between embryo axis and cotyledon were consistent between the qRT-PCR and the Illumina transcriptome analyses. For example, unigene70917, annotated as late embryogenesis abundant 3 (LEA3) protein (Figure 5B), unigene36175 annotated as ATS/KAN4 transcription factor (Figure 5C) and unigene 72799, annotated as cyclopropane fatty acid synthase (Figure 5D) appeared to express only in the embryo axis, with little if any, expression in cotyledons as indicated by the transcriptome analysis. Such an expression pattern was also validated by qRT-PCR that indicated little or no expression of these two genes in root and leaf tissues. Conversely, unigene4514, annotated as lipid binding protein (Figure 5E), unigene37407 annotated as RING-H2 finger protein (Figure 5F) and unigene25168 annotated as isocitrate lyase (Figure 5G) appeared to express almost exclusively in cotyledons as indicated by both transcriptome and qRT-PCR analyses. There are also genes with similar expression levels in both embryo axis and cotyledon, as represented by both transcriptome and qRT-PCR analyses of unigene29210, annotated as polyadenylate-binding protein 2 (Figure 5H) and unigene19199, annotated as uridine 5′-monophosphate synthase (Figure 5I). The qRT-PCR analysis of these two genes also indicated that they have comparable levels of expression in both root and leaf tissues, in addition to developing seeds (Figures 5H and 5I). In addition, qRT-PCR validations of the other 21 selected unigenes were also found to be consistent with the transcriptome analysis (Figure S1).

### Differential Expression of Transcription Factors in Cotton Embryo

Zygotic embryogenesis and seed development is the result of a suite of distinct gene expression programs that are precisely coordinated in higher plants [10]. Transcription factors (TFs) are a class of special proteins that can regulate gene expression at transcription level. A total of 2,097 unigenes annotated as TFs were identified in the current cotton embryo transcriptome, which can be classified into 121 families. The top five TF families were identified as bHLH domain, MYB-related, AP2, bZIP and WRKY domain in terms of sequence abundance (Table S2). For comparing TF expression levels between cotyledon and embryo axis, we extracted TFs with RPKM values >100 in either libraries, of which 17 showed more than 2-fold higher expression in embryo axis than in cotyledon and 44 showed more than 2-fold higher expression in cotyledon than in embryo axis (Table S2).

At the middle maturity stage of cotton seed development, the cotyledons and embryo axis are differentiated into nutrient storage and embryo organs. The expression of TFs might have moved from the key roles in determining polar differentiation and embryo formation towards metabolism of lipids, protein, carbohydrate, and secondary metabolite products. This might be reflected in the relatively low level of expression of TFs such as SHOOT MERISTEMLESS (STM), WUSCHEL-related homeobox (WOX) and PIN-formed (PIN) TF genes that are normally highly expressed in the early stage of embryo development in Arabidopsis [11]–[13]. The STM gene, encoded by a class I KOTTED-like homeodomain-containing protein is required for shoot apical meristem formation during embryogenesis and is expressed in only a specific set of cells within the embryo apex [11]. RNA-seq data shows that 22 unigenes were annotated as KOTTED-like class I KOTTED-like homeodomain-containing protein, half of which (11 uniques) displayed low level yet specific expression in embryo axis, while the other half demonstrated low level yet cotyledon-specific expression (Table S2). The expression of WOX is necessary for cell divisions that form the apical embryo domain [12]. Transcriptome analysis revealed that some unigenes encoding the WOX transcription factors, such as unigene68834 and unigene69004, had moderately higher expression in embryo axis compared to the KOTTED-like proteins, although its expression in cotyledon is also very low. This might indicate that moderate expression level of WOX TFs is still necessary at mid-maturity seed development (Table S2). *PIN* genes encode transporter-like membrane proteins that are important for regulating auxin transport and mutations in *PIN1* and *PIN7* disrupt the establishment of the embryogenic apical-basal axis in Arabidopsis [13]. Our results showed that two unigenes including unigene68257 (RPKM 81.90) and unigene65306 (RPKM 88.86) annotated as PIN transporters were specifically expressed in embryo axis, but absent in cotyledon, implying that in the mid-maturity stage of cotton embryo development, regulation of auxin transport is still important to embryo axis development but less so in cotyledon. Perhaps most surprisingly, the axial regulator YABBY encoded by 10 unigenes in cotton demonstrated strong cotyledon-specific expression (Table S2). In Arabidopsis, *YABBY* genes were mostly expressed in lateral organ primordial produced from the apical and flower meristems [14]–[16].

Several transcription factors such as LEAFY COTYLEDON1 (LEC1), LEAFY COTYLEDON2 (LEC2), FUSCA3 (FUS3) and abscisic acid insensitive3 (ABI3) have been identified as master regulators of seed development and maturation and the ectopic expression of each of the *LEC1, LEC2* and *FUS3* genes could make the vegetative and reproductive tissues to adopt characteristics of maturation phase embryos [17]. As shown in Table S2, in both the cotyledon and embryo axis, the expression of *FUS3* was significantly higher than those of *LEC1* and *LEC2*. *LEC2* showed 1.25 fold higher expression in embryo axis than cotyledon, while both the *FUS3* and *LEC1* were expressed equally between embryo axis and cotyledon. Twelve unigenes annotated as *ABI3* were identified and among them 3 unigenes (unigene3165, unigene20058 and unigene23620) showed significantly higher expression in cotyledon than in embryo axis (Table S2). In Arabidopsis, LEC1 and WRI1 have been identified as two key transcription regulators involved in oil accumulation [18] and the overexpression of either *LEC1* or *WRI1* resulted in oil increase without any negative impacts on the grain yield [19]. In transcriptome analysis, one unigene was annotated as *WRI1* transcription factor that showed significantly higher expression (RPKM value 107.01) in cotyledon than in embryo axis: (RPKM value 33.29) (Table S2). This is consistent with the relatively higher oil accumulation in cotyledons compared to embryo axis [20].

### Transcriptome Analysis of Carbohydrate Metabolism in Developing Cotton Seed

Although there is little or no starch in the mature cotton seed, starch biosynthesis and degradation become active at the early stage of embryo development and reach maximal level at the early-mid stage of development. ADP-glucose pyrophosphorylase (AGPase) as a key enzyme catalysing the first committed step of starch biosynthesis was represented by 23 unigenes. Total RPKM values for them indicated that high expression of AGPase also happened in 30 DAP cotton embryo and 0.75 fold more expression in embryo axis than in cotyledon. Forty unigenes were annotated as starch synthase that could be classified into several subgroups, including the soluble starch synthase I, II, III, granule-bound starch synthase I, II, starch synthase IV, V, and starch synthase-like protein (Table S3). The evaluation of RPKM values indicated that starch synthases were highly expressed in 30 DAP cotton embryo and had 0.3 fold on average higher expression in embryo axis than in cotyledon. Among them, transcripts encoding soluble starch synthase III and starch synthase-like protein ATSS4 showed the highest expression. A number of key enzymes involved in starch degradation including amylase, alpha-glucan water dikinase (GWD) and 4-alpha-glucanotransferase (DPE) in cotyledon and embryo axis tissues (Table S3) showed relatively low, yet similar expression levels in the embryo axis and cotyledonary tissues.

Instead of starch, cotton seed accumulates complex sugars in the form of galactosides including raffinose and stachyose up to 10% of the total seed dry weight in a mature cotton seed [21]. A set of galactosyltranferases is involved in the biosynthesis of these oligosachharides [22]. Galactinol synthase (GYG) catalyses the synthesis of galactinol from UDP-galactose and myo-inositol. Raffinose is synthesised by the transfer of galactosyl residue from galactinol to sucrose by raffinose synthase (RFS), while a second galactose addition produces stachyose by stachyose synthase (STS). RNA-seq data revealed that 5, 21 and 5 unigenes were annotated as GYG, RFS and STS, respectively (Table S4). Further analysis of total RPKM values for galactosyltransferases demonstrated that GYG, RFS and STS generally had higher expression in embryo axis than in cotyledon, at 0.90 fold, 2.13 fold and 0.84 fold, respectively, indicating that galactosides synthesis may be more active in embryo axis than in cotyledon of a mid-maturity cotton seed (Table S4). Further, the substantially higher RPKM value of RFS than those of GYG and STS may explain why raffinose is the predominant soluble sugar in cotton seed [23].

Sucrose provides the carbon skeleton for cotton seed oil by the reaction of sucrose synthase (Sus) and invertase. There were almost equivalent levels of *Sus* in embryo axis (RPKM 1314.67) and cotyledon (RPKM 1347.47), which were more than 1.5 fold higher than that for alkaline/neutral invertase (*A/N-INV*) (Table S3). The expression of other two types of invertases, including cell-wall invertase (*CW-INV*) and vacuolar invertase (*VC-INV*) were even lower with RPKM value below 30 (Table S3). This is consistent with previous studies that Sus activity was about five fold higher than VC-INV and more than 10-fold higher than A/N-INV in developing cotton embryos [24]. Similarly, in some other oilseeds, such as *B. napus*, *Ricinus communis*, *Euonymus alatus* and *Tropaeolum majus*, *Sus* ESTs were 20–40 folds higher than neutral invertases [25].

The sucrose derivatives are utilised through both cytosolic and plastidic glycolytic pathways. Multiple transporters, including glucose-6-phosphate translocator (GPT), triose phosphate translocator (TPT), and phosphoenolpyruvate translocator (PPT), facilitate the exchanges between cytosol and plastid for the intermediates that are generated during glycolysis [26]. The expression of *PPT/TPT* and *GPT*, represented by RPKM values, indicated that these transporters were highly expressed in 30 DAP cotton embryo and had little expression variation between cotyledon and embryo axis (Table S3). This is consistent with a previous study on the transcriptome of mesocarp of oil palm [27].

### Transcriptome Analysis of Genes Involved in *de novo* Fatty Acid Biosynthesis in Developing Cotton Seed

**Figure 6:**
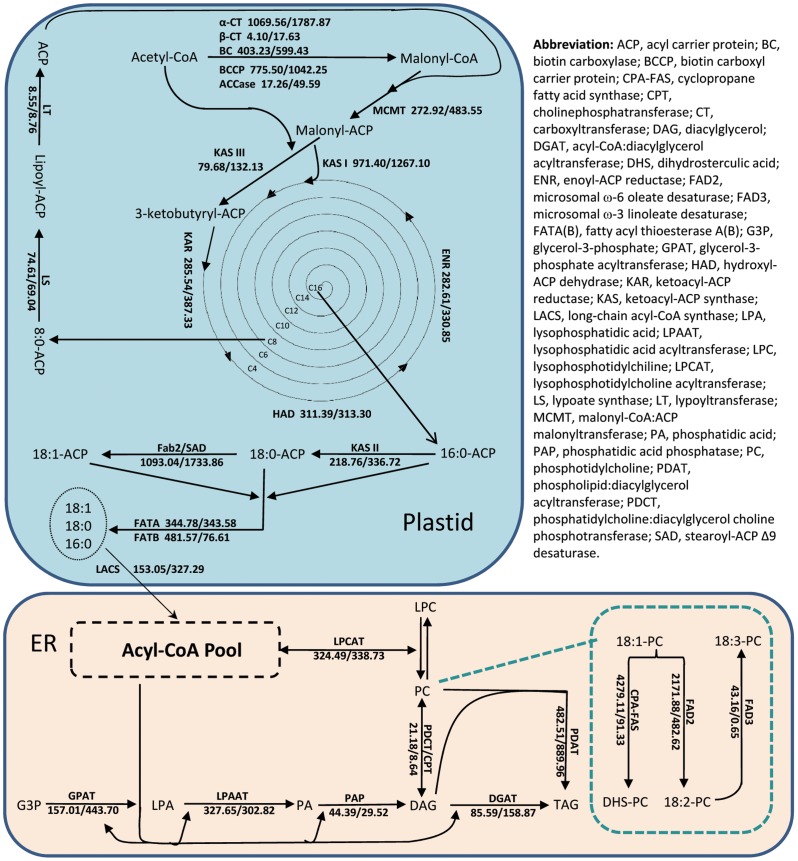
RPKM of unigenes in the transcriptome analysis for genes encoding enzymes in the fatty acid, TAG and oil body biosynthetic pathways in 30 DAP cotton embryo axis and cotyledon. The numbers after the gene (a/b) are the RPKM values for the corresponding genes in the transcriptome analysis of cDNA libraries: a, embryo axis; b, cotyledon.

At the first metabolic step of *de novo* fatty acid biosynthesis in plastid, Acetyl-CoA Carboxylase (ACCase) catalyses the addition of one carboxyl group to the acetyl-CoA and then forms malonyl-CoA (Figure 6). Interestingly, the unigenes encoding subunits of heteromeric ACCase (α-CT,BCCP and BC) showed significantly higher expression than the subunits of homomeric, multiple-functional ACCase (Fig. S1). The malonyl group is then transferred from CoA to an acyl-carrier protein (ACP) that serves as the carrier for the growing fatty acyl chain. Malonyl-ACP is extended to a four-carbon molecule by the second acetyl-CoA condensing enzyme, ketoacyl-ACP synthase III (KASIII). The repeated process of adding two-carbon units onto the elongated fatty acid chain is catalysed by KASI leading to the formation of palmitoyl-ACP (C16∶0-ACP). KASII catalyses the elongation of palmitoyl-ACP to stearoyl-ACP (C18∶0-ACP). It appears that the expression of each of the three KAS enzymes (KASI, II and III) showed no significant difference of RPKM values between cotyledon and embryo axis, despite the expression of KASI was significantly higher than KASII and KASIII. Among all the key genes involved in fatty acid biosynthesis in plastids, the soluble stearoyl-ACP Δ9-desaturase (SAD) that is responsible for the introduction of the first double bond onto Δ9 position of C18∶0-ACP to form C18∶1-ACP showed the highest RPKM values (Table S5). Unigene82132 that had the highest expression among all the 15 SAD unigenes showed 1.85 fold higher expressions in embryo axis than in cotyledon (Table S5). In contrast, unigene67165 showed nearly 2.3 fold higher expression in cotyledon compared to embryo axis. Such a cotyledon preferential expression pattern was also observed in other SAD-encoding unigenes, including Unigene63978, Unigene74306, Unigene67165, Unigene63978, Unigene74306, Unigene3726 and Unigene67165 (Table S5). Finally, the fatty acid biosynthesis will be terminated by acyl-ACP thioesterases (FatA and FatB). The evaluation of RPKM values of *FatA* and *FatB* indicated that both the acyl-ACP thioesterase genes had basically consistent expression between embryo axis and cotyledon (Table S5).

### Transcriptome Analysis of Membrane-bound Lipid Modifying Enzymes

**Figure 7:**
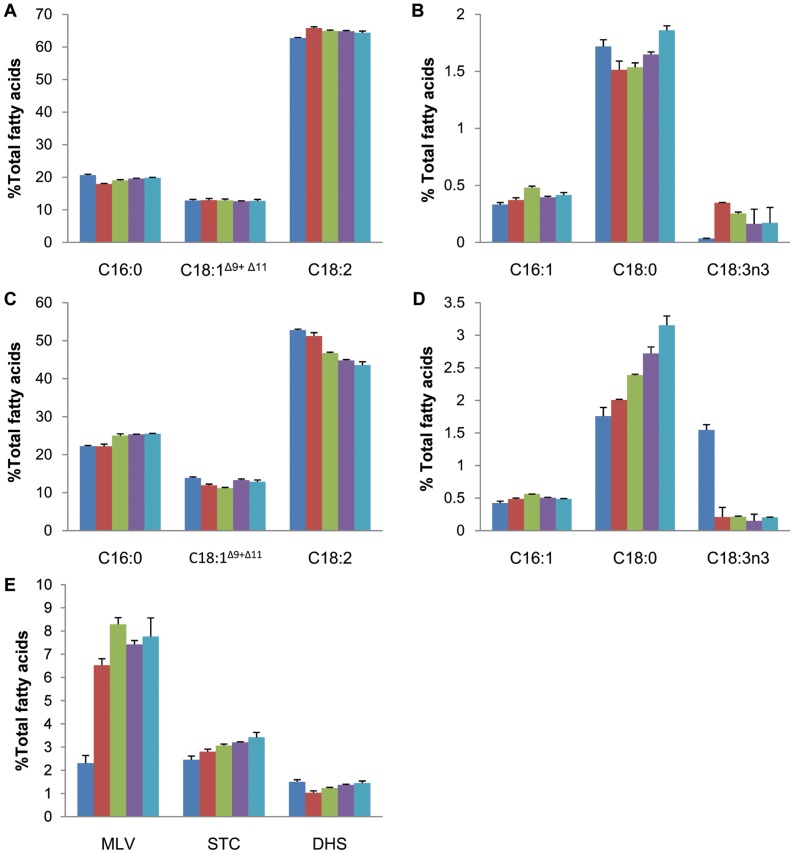
Fatty acid composition of total lipids from developing cotton embryo axis and cotyledon. A, major fatty acids in cotyledon; B, minor fatty acids in cotyledon; C, major fatty acids in embryo axis; D, minor fatty acids in embryo axis; E, carbocyclic fatty acids in embryo axis. Results represent the mean SD of three biological replicates. Minor fatty acid (accounting for <1% of the fatty acid composition) are not shown. C16∶0, palmitic acid; C16∶1, palmitoleic acid; C18∶0, stearic acid; C18∶1 (mainly C18∶1^Δ9^, oleic acid, with a small amount of C18∶1^Δ11^, *cis*-vaccenic acid); C18∶2, linoleic acid; C18∶3^n3^, α-linolenic acid; MLV, malvalic acid; STC, sterculic acid; DHS, dihydrosterculic acid. Analysed samples were derived from developing cotton embryos of 25 DAP (blue), 30 DAP (red), 35 DAP (green), 40 DAP (purple), and 45 DAP (cyan).

On ER membranes, oleic acid becomes associated with phosphatidylcholine (PC) and can be further modified by a microsomal ω-6 fatty acid desaturase FAD2 to form linoleic acid that accounts for more than 50% of total fatty acids in cotton seed oil (Figure 6). We have analysed the fatty acid composition of developing cotton embryos ranging from 25 to 45 DAP, harvested at 5 days intervals. In cotyledonary tissues, as shown in Figure 7A, linoleic acid (C18∶2^Δ9,12^) accounts for the highest proportion of total fatty acids, at about 60∼65% while palmitic acid (C16∶0) was present at 18∼20%, followed by oleic acid (C18∶1^Δ9^) and its Δ11 isomer at ∼12%. Three minor fatty acids, including palmitoleic acid (C16∶1^Δ9^), stearic acid (C18∶0) and α-linolenic acid (C18∶3^Δ9,12,15^) were each present at <2% (Figure 7B). Interestingly, there was little variation in fatty acid composition of cotyledon at all the five consecutive developmental stages. Such an observation suggests that the biosyntheses of fatty acids in developing cotton cotyledons could reach the balanced final composition as early as 25 DAP and it does not vary significantly at subsequent developmental stages. Similar to cotyledon, as shown in Figure 7C, linoleic acid also accounts for the highest proportion of total fatty acids present in embryo axis, at about 45∼53%; palmitic acid was present at 20∼25%, followed by oleic acid at 10∼12%. The other three relatively minor fatty acids, including palmitoleic acid, stearic acid and α-linolenic acid were each present at <3.5% in the embryo axis of developing cotton embryos (Figure 7D).

This study on comparative transcriptome analysis of cotton developing seed identified two members including *FAD2-1* and *FAD2-2*, of which the former was equally expressed between cotyledon and embryo axis, while the latter was preferentially expressed in embryo axis with 5.19 fold higher expression in embryo axis than cotyledon (Table S5). It has been previously demonstrated that *FAD2-1* has the capability of converting about 80% oleic acid into linoleic acid in developing cotton seed [28]. Molecular analysis of gene expression suggested that the *FAD2-1* is specifically expressed in the developing seeds and its expression reaches the highest level at the mid-maturity stage of seed development, between 25–35 DPA, while drastically declining when seeds approach maturity at 45 DPA [29]. *FAD2-2* cDNA sequence was previously reported as a constitutively expressed microsomal ω-6 fatty acid desaturase [30]. However, this study revealed its preferential expression in embryo axis, which was not reflected in whole seed analysis in previous studies.

**Figure 8:**
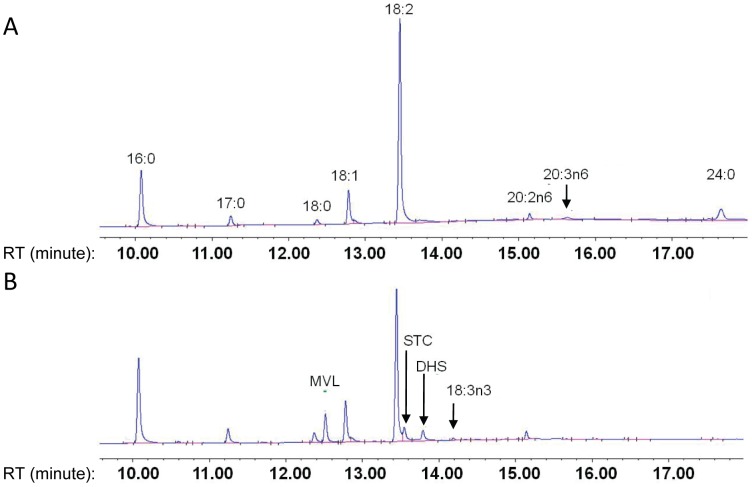
GC analysis of FAMEs extracted from developing embryo. (A) 30 DAP cotyledon (B) 30 DAP embryo axis.

Transcriptome data revealed that unigene69299 and unigene66043 were annotated as microsomal ω-3 fatty acid desaturase (*FAD3*) and both of them with rather low RPKM values, but with six fold higher in embryo axis than in cotyledon (Table S5). Such a preferential expression pattern of *FAD3* in embryo axis is also in agreement with the analysis of fatty acid composition that demonstrated higher α-linolenic acid accumulation in embryo axis than cotyledon (Figure 8). Seven unigenes with low RPKM values in both the embryo axis and cotyledon libraries were identified as fatty acid elongases (*FAE*) and they all showed about 1 fold higher expression in cotyledon than in embryo axis (Table S5). This is also consistent with the exclusive presence of elongated fatty acids including C20∶3 and C24∶0 in cotyledons (Figure 8).

In addition to acyl-PC modification on ER, acyl lipids located in the plastid envelope could also be further modified by chloroplast envelope-bound or thyiakoid-bound fatty acid desaturases, such as FAD6, FAD7 and FAD8 [31]. The plastidial ω-6 fatty acid desaturase FAD6 encoded by unigene19739 showed equally low expression in both embryo axis and cotyledon (Table S5). The plastidial ω-3 fatty acid desaturase is encoded by *FAD7* and *FAD8*. *FAD7* showed equally low expression in both embryo axis and cotyledon, in contrast to *FAD8* that showed 3 fold higher expression in embryo axis relative to cotyledon (Table S5).

Perhaps the most striking difference between a cotyledon and embryo axis, in term of fatty acid composition, is the exclusive presence of the three major carbocyclic fatty acids including dihydrosterculic acid (DHS), sterculic acid (STC) and malvalic acid (MVL), together accounting for 6∼12% of total fatty acids in embryo axis at 25–45 DAP (Figure 7E). Compared to cotyledonary tissues, the unique presence of carbocyclic fatty acids in embryo axis is mainly at the expense of linoleic acid level. For instance, linoleic acid level in embryo axis at 35 DAP is only about 45% of total fatty acids compared to 65% in cotyledons. This is somewhat anticipated as the biosyntheses of carbocyclic fatty acids competes oleic acid substrate with the biosynthesis of linoleic acid [32]. It is also worthy of noting that the accumulation of linoleic acid in embryo axis tends to decrease as the embryo development progresses (Figure 7C). This is generally correlated with the increased levels of carbocyclic fatty acids and saturated fatty acids, i.e palmitic acid and stearic acid. Compared to cotyledonary tissues, there is an approximate 25% increase of palmitic acid, and more than 50% increase of stearic acid in embryo axis across the five consecutive developmental stages. The oleic acid accumulation is relatively consistent between these two embryo tissues. The increase of saturates in embryo axis might be the result of the suppression of fatty acid desaturation by STC and MVL as previously reported in mammals as well as other organisms [33].

A fatty acid methyltransferase, known as cyclopropane fatty acid synthase (CPA-FAS), was shown to convert oleic acid to DHS using *S-*adenosyl methionine as the methyl donor [28], [34]. As indicated by its RPKM value, unigene72799 encoding for cotton CPA-FAS was among the most highly expressed genes in embryo axis transcriptome, with 8.06 fold higher expression compared to cotyledon (Table S5).

### Expression of TAG Assembly Enzymes in Cotton Seed

The fatty acids formed in the acyl CoA pool may be incorporated into membrane and storage lipids *via* the Kennedy pathway by the sequential esterification of glycerol-3-phosphate through the action of glycerol-3-phosphate acyltransferase (GPAT) to form lysophosphatidic acid, followed by 1-acyl-sn-glycerol-3-phosphate acyltransferase (LPAAT) to form phosphatidic acid (PA). Dephosphorylation of PA by phosphatidic acid phosphatase results in the formation of diacylglycerol (DAG), which is then acylated to form TAG by acyl-CoA:diacylglyerol acyltransferase (DGAT) [35]. Transcriptome data revealed that GPAT6 was encoded by 4 unigenes and GPAT9 was encoded by 3 unigenes in developing cotton embryos at 30 DAP. The RPKM values of the unigenes encoding for GPAT6 were approximately 10 fold more in cotyledon than in embryo axis (Table S5). Since GPAT6 was found to play a key role in cutin biosynthesis [36], it is likely that higher level of cutin biosynthesis occurs in cotyledon than in embryo axis. In contrast, *GPAT9* showed ubiquitously low expression in both tissues. Similarly, the RPKM values of *LPAAT, DGAT1, DGAT2* unigenes were also rather low and consistent between cotton embryo axis and cotyledon (Table S5).

Phosphatidylcholine:diacylglycerol cholinephosphotransferase (PDCT) catalyses the inter-conversion of PC and DAG by transferring the phosphocholine head group between these two molecules (Figure 6) [37]. The RPKM value of *PDCT* showed 1.31 fold higher in embryo axis than in cotyledon (Table S5). This is somewhat unexpected as the higher expression of *PDCT* should lead to higher level of polyunsaturated fatty acid as it could increase the availability of PC substrate to FAD2 and FAD3 enzymes. However, cotton embryo axis contains relatively less polyunsaturated fatty acids compared to cotyledons (Figure 7). TAG can also be formed in plants *via* an acyl-CoA independent pathway catalysed by phospholipid:diacylglycerol acyltransferase (PDAT) [38]. PDAT catalyses the transfer of an acyl group from the *sn-*2 position of PC to the *sn*-3 position of DAG and yields TAG [38]. Transcriptome analysis revealed that 15 unigenes were annotated as PDAT1, and none of which showed significantly different expression levels between embryo axis and cotyledon (Table S5).

### Expression of Oleosin in Cotton Seed

Oleosin is a class of small proteins associated with the oil body membrane in plant seeds and it is known to play dual physiological roles, acting as protectors for stabilising the oil bodies in developing seeds and mature seeds, and as the recognition signal for lipase binding in germinating seeds [39]. Two distinct oleosins MatP6 and MatP7, 77% identical to each other, were found to express during the maturation and post-abscission stages of cotton embryogenesis [40]. Transcriptome data revealed that 13 unigenes were annotated as oleosins and among them unigene76029 annotated as *MatP6-A* had the highest expression by RPKM values (621.84 in embryo axis and 361.92 in cotyledon), showing nearly 1 fold higher expression in embryo axis than cotyledon (Table S5). In contrast, other oleosin unigenes showed relatively low RPKM values ranging from 0.66 to 122.51, and only trivial variation between cotyledon and embryo axis (Table S5). Steroleosin, a minor integral oil body protein that has been found in Arabidopsis and sesame seed oil bodies in minute quantities [40], was highly expressed in cotton embryos, represented by unigene47835 with RPKM values of 541.68 in cotyledon that was ∼1.3 fold higher than in cotyledon (RPKM 222.98) (Table S5).

### Expression of Lipid Transfer Proteins and Late Embryogenesis Abundant Proteins

Lipid transfer proteins (LTPs) with different sizes are widely distributed in various tissues of a higher plant and are involved in multiple biological process, such as defence against bacterial and fungal pathogens, adaptation to stressful environmental conditions, pollen tube adhesion to the transmitting tract of the style, and export of cuticular waxes [41]. In cotton, *LTPs* were found specifically expressed in the elongating cotton fibre in a temporal manner, indicating direct contribution to the elongation and development of fiber cells [42]. Forty-one unigenes were identified as *LTPs* in the developing cotton embryos, among them 17 unigenes were up-regulated and 24 unigenes were down-regulated in embryo axis (Table S6). Unigene37407 is the most highly expressed *LTP* and is among the 100 most abundant unigenes in cotyledon (1,012.35 RPKM). It showed 4.18 fold higher expression in cotyledon than in embryo axis (55.89 RPKM) (Table S6). The biochemical significance of the differential expression of *LTPs* in these two embryo tissues remained to be explored.

According to Hughes and Galau, as many as 18 different late embryogenesis abundant (LEA) proteins could be up-regulated in late embryogenesis stage in cotton as the seeds approach maturity [43]. It is hypothesised that some of these LEA proteins are functionally involved in eliciting desiccation tolerance in the seed and their synthesis is correlated with abscission of the funiculus that terminates nutrition and water transport to the seed from the mother plant [43], [44]. In this study, 13 unigenes encoding 8 LEA proteins including LeaD-11, Lea14, LeaB19.4, Lea19, Lea34, Lea5D, Lea5A and Lea29 were highly expressed in both seed tissue types and all showed preferable expression in embryo axis (Table S6). This might indicate that the desiccation involving LEA protein had already begun in cotton developing embryos as early as 30 DAP embryo, especially in embryo axis.

### Differential Expression of Seed Storage Proteins in Developing Cotton Embryos

In cotton seed, the two major classes of storage proteins including globulins and albumins that differ in their solubility properties are synthesised and compartmentalised in storage protein vacuoles during cotton seed maturation [45]. Globulins can be further classified based on the sedimentation rate of their aggregated forms into the 7S vicillins and 11/12S legumins [46]. In a recent survey vicillins and legumins families comprises 60–70% of the total cotton seed proteins [47]. Two distinct members of the vicillin family, Vicillin A and Vicillin B that share 72% amino acid similarity, represent the first discovered cotton seed storage proteins [48]. Similarly there are two legumin isomers, Legumin A and Legumin B that are more diverged compared to the vicillin gene family, sharing only 58.5% similarity in amino acid sequences. The current transcriptome analysis revealed that genes encoding these seed storage proteins were the most highly expressed in the entire transcriptome of cotton embryo, represented by 25.66% of the total RPKM value in cotyledon and 22.71% in embryo axis (Table 2). Interestingly, *2S Albumin* transcripts were more abundant compared to either legumins or vicillins, accounting for 11.63% of total RPKM value in cotyledon and 9.96% in embryo axis in this study (Table 2). This is in contrast to a recent proteomic analysis on cotton seed storage protein revealing that vicillins and legumins, rather than 2S albumin, constituted the majority of cotton seed proteome [47]. The discrepancy between transcriptomic and proteomic analyses of 2S albumin might imply that it is subjected to both transcriptional and translational regulations.

**Table 2:** The summary of comparative transcriptome analysis of seed storage proteins.

Storage protein	Derivation of subgenome	Gene ID	COT_RPKM	Percent of total RPKM in COT	EAX_RPKM	Percent of total RPKM in EAX
2S albumin	unknown	gi|167310|	283156.23	11.63%	243409.27	9.96%
Legumin B	D-subgenome	gi|167372|	75475.81	3.10%	44625.58	1.83%
	A-subgenome	gi|346426301|	9.8934	–	7.1475	–
Legumin A	D-subgenome	gi|167376|	52570.78	2.16%	38203.54	1.56%
	A-subgenome	gi|346426292|	1.389	–	3.0965	–
Vicilin A	D-subgenome	gi|346426313|	45876.48	1.88%	49495.79	2.02%
	A-subgenome	gi|346426309|	131.8682	–	183.545	–
Vicilin B	D-subgenome	gi|167374|	167713.94	6.89%	179399.62	7.34%
	A-subgenome	gi|346426321|	104.876	–	100.989	–
**Total**			624793.25	25.66%	555133.79	22.71%

Note: Cotyledon and embryo axis were abbreviated as COT and EAX, respectively.

*Vicillin B* showed 1.58 fold higher expression than *Vicillin A* in both embryo axis and cotyledon. This is consistent with proteomic analysis that Vicilin B protein was ∼25% more than Vicillin A in cotton seed [47]. Similarly, *Legumin B* had 0.52 and 0.22 fold higher expression than *Legumin A* in cotyledon and embryo axis, respectively (Table 2). Cotton is an allotetraploid species originated from an ancient hybridisation between A-genome diploid cotton related to *G. herbaceum* and D genome diploid cotton related to *G. raimondii* [49]. Biased accumulation of Legumin B of D-subgenomic origin, likely due to the concerted evolution between the two subgenomes, was recently documented by proteomic analysis in cotton [47]. Such a trend was confirmed in the transcriptional level in this study. D-genome-derived *Legumin B* demonstrated rather high expression at transcription level by RPKM values: 75,475.81 in cotyledon and 44,625.58 in embryo axis, which is significantly higher than A-genome-derived *Legume B* with RPKM values: 9.8934 in cotyledon and 7.1475 in embryo axis (Table 2).

### Gossypol Metabolism

**Figure 9:**
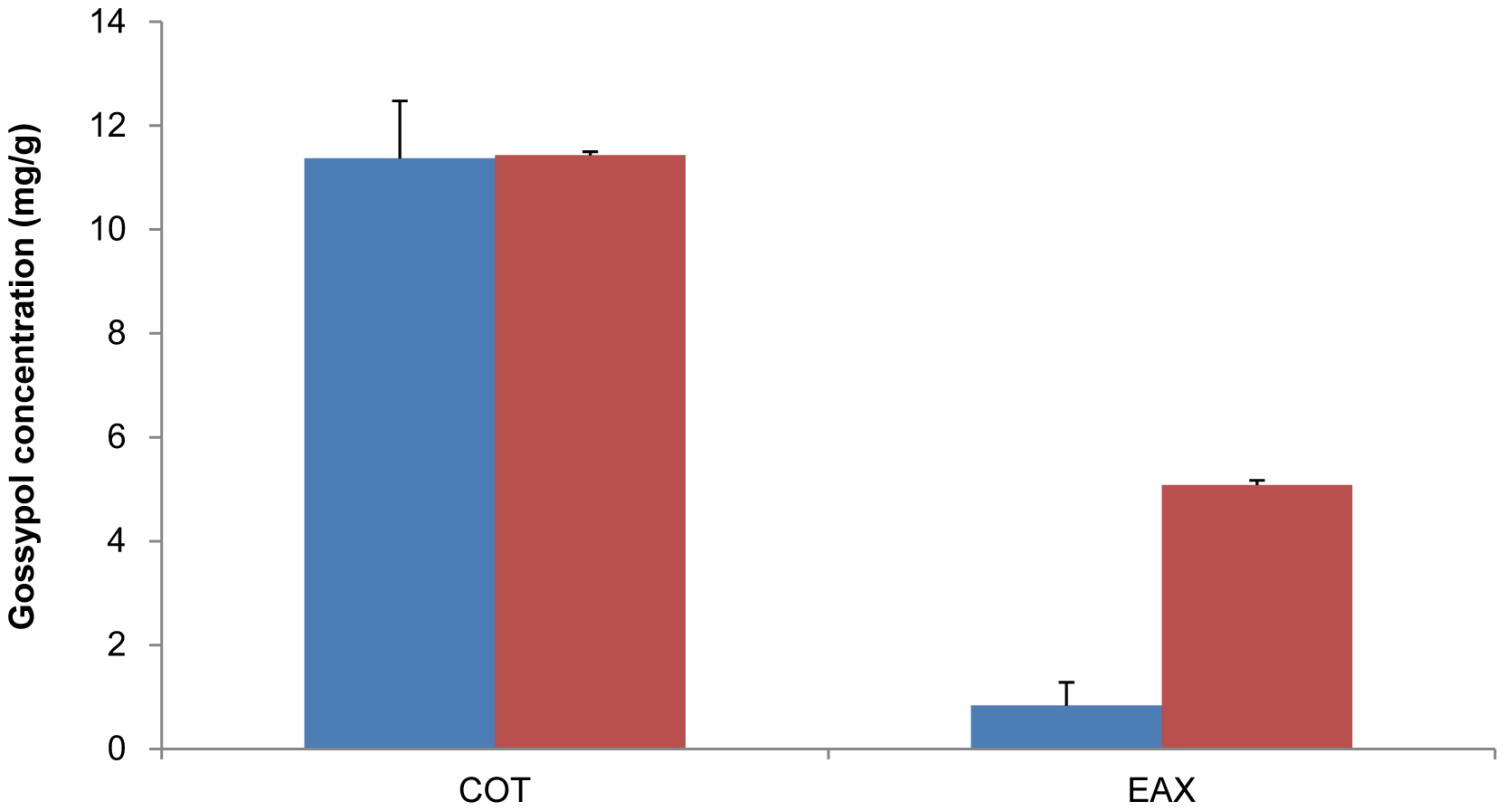
Gossypol content in cotton embryo axis and cotyledon. Cotyledon and embryo axis abbreviated as COT and EAX, respectively. Analysed samples were from: developing cotton embryo (cyan) and mature cotton seed (red).

Cotton is characterised by the presence of gossypol in lysigenous glands of cotton plants, including cotton seeds, which are sesquiterpenes derived from a cytosolic branch of terpenoid metabolism *via* the mevalonate pathway [50]. Farnesyl diphosphate (FPP) is generated as the linear carbon skeleton of the sesquiterpenes and its cyclisation catalysed by a terpene cyclase enzyme, (+)-δ-cadinene synthase, to form (+)-δ-cadinene is the first committed step in gossypol biosynthesis [51]. (+)-δ-cadinene is then hydroxylated at the C-8 position leading to 8-hydroxy-(+)-δ-cadinene, through the action of cytochrome P450 enzyme (+)-δ-cadinene-8-hydroxylase (CYP706B1). Subsequently 8-hydroxy-(+)-δ-cadinene is converted to desoxyhemigossypol (dHG) and further oxidised by one electron into hemigossypol prior to the formation of gossypol by phenolic oxidative coupling [52]. Transcriptomic analysis demonstrated that two key enzymes, (+)-δ-cadinene synthase (unigene37248) and (+)-δ-cadinene-8-hydroxylase (unigene35113) showed 3.2 and 2.7 fold higher expression in cotyledon than in embryo axis, respectively (Table S7). The unigenes encoding the enzymes involved in gossypol biosynthesis pathway showed generally higher expression in cotyledon than in embryo axis according to RPKM values. Such a finding is consistent with preferential accumulation of gossypol in cotyledon compared to embryo axis (Figure 9).

### Conclusions

The cotton embryos at the mid-maturity stage is characterised by an increase in the size and weight of the cotyledons and rapid accumulation of oil and storage proteins that are the major reserves of carbon and nitrogenous compounds needed for seed germination and early seedling growth. In this study we have provided a comprehensive dataset that documents the dynamics of the transcriptome at the mid-maturity stage of cotton seed development and in discrete seed tissues, including embryo axis and cotyledon tissues. The results showed that cotton seed is subject to many transcriptome variations in these two tissue types, enabling the identification of a great deal of differentially expressed genes related to seed development, lipid, protein, carbohydrate metabolism and secondary metabolism. The comparative expression profiling strategy between embryo axis and cotyledon provided a subset of genes that were differentially expressed. The differential gene expression between cotton embryo axis and cotyledon uncovered in our study should provide an important starting point for understanding how gene activity is coordinated during seed development. Further, the identification of genes involved in rapid metabolite accumulation stage of seed development will extend our understanding of the complex molecular and cellular events in these developmental processes and provide a foundation for future studies on the metabolism, embryo differentiation of cotton as well as other dicot oilseed crops.

While globally the process of development and metabolism in cotton seed adheres closely to that exhibited by the Arabidopsis seed, some interesting cotton-specific features have emerged from this transcriptome study. An area meriting more intensive study is the exclusive accumulation of carbocyclic fatty acids in embryo axis. Genes involved in fatty acid and lipid biosynthesis are among the most regulated in these two seed tissue types, suggesting specialised function in seeds for these genes. The information derived from the transcriptomics dataset could be used to further investigate whether differential expression between these two tissues is specific to carbocyclic fatty acid biosynthetic enzymes or holds true more generally for enzymes involved in the biosynthesis of other fatty acids as well.

## Materials and Methods

### Growth Condition of Plant and Collection of Embryo Samples

Upland cotton (*Gossypium hirsutum* L.) cv. Coker-315 was grown under greenhouse conditions with 16 h photo period and constant temperature at 28°C, 50% humidity, and 600 µmol m^−1^ s^−1^ light intensity. Embryo axis and cotyledon were dissected from seed of fruits harvested at 25 DAP, 30 DAP, 35 DAP, 40 DAP, and 45 DAP, immediately frozen in liquid nitrogen and then stored at −80°C until use for RNA isolation and lipid analysis. Total RNAs were extracted from 30 DAP cotyledon and embryo axis separately using the Plant RNeasy Kit (Qiagen, Hilden, Germany) according to the manufacturer’s instructions. Total RNAs from cotton leaves and roots were isolated as previously described [53]. Briefly, 200 mg fresh leaves or root were ground in 2 mL RNA extraction buffer containing 30 mM EGTA, 1% SDS, 1% Sodium deoxycholate, 2% PVP-40, 0.5% NP-40 and 10 mM DTT, supplemented by 60 µL 25 mg/mL Proteinase K. The flow-through of Shredder Columns was mixed with 0.5 volume absolute ethanol and loaded onto RNeasy mini columns and washed by adding 350 µL RW1 (Qiagen). RNAs were eluted with 50 µL RNase-free water and the concentration of each RNA sample was determined spectrophotometrically and the integrity of all RNA samples was monitored on agarose gel.

### RNA Sample Preparation and Massively Parallel Sequencing

Twenty µg of total RNA was prepared from cotton cotyledon or embryo axis derived from mid-maturity developing cotton embryos for Illumina sequencing. Magnetic beads with poly(dT) oligos attached were used for purifying the mRNA from the total RNA. The Fragmentation buffer was then added for interrupting mRNA to short fragments. Using these short fragments as templates, random hexamer-primer was used to synthesise the first-strand cDNA (Invitrogen, Carlsbad, CA, USA). The second-strand cDNA was synthesised using RNaseH and DNA polymerase I (Invitrogen). Following separation on the agarose gel electrophoresis, the DNA fragments more than 300 bp in length were selected for the PCR amplification as templates. At last, the libraries were sequenced in Beijing Genome Institute (BGI, Shenzhen, China) using an Illumina GA IIX following the manufacturer’s protocol.

Following the deletion of the empty reads, the adaptor sequences, and the low-quality sequences, the clean reads were assembled into contigs and scaffolds based on pair-end information using SOAPdenovo (http://soap.genomics.org.cn/soapdenovo.html). With the Blast2GO program [54], functional annotation of the unigenes was performed by carrying out BLAST (E-value<1.0e^−5^) against NCBI Nr, COG (http://www.ncbi.nlm.nih.gov/COG) and KEGG (http://www.genome.jp/kegg/) databases. To estimate the expression level of each unigene, the mapped read counts for each gene were normalised for the total read number in the lane according to Reads Per kb per Million reads (RPKM).

### Real Time qRT-PCR Analysis

The first strand cDNA was prepared from 500 ng total RNAs derived from cotton root, leaf, embryo axis and cotyledon using First Strand cDNA Synthesis Kit (OriGene Inc., Australia) according to manufacturer’s instructions. Gene-specific primers were designed based on the gene sequences using online software Primer3 (Table S8). Real time qRT-PCR was performed with BIO-RAD CFX^TM^96 (BIO-RAD, Hercules, CA, USA) in a final volume of 10 µL containing 5 µL of 2x iQ™ SYBR®Green Supermix (BIO-RAD), 2 µL of cDNA, 10 µM of forward and reverse gene specific primers. The thermal cycling conditions were as follows: 95°C for 3 min for denature, 40 cycles at 95°C for 10 s, 60°C for 30 s, and 68°C for 30 s. The *Uniquitin14* gene was used to normalise gene expressions. The relative changes in gene expression levels were calculated using the Bio-Rad CFX manager.

### Lipid Extraction and Fatty Acid Analysis

The total lipids from cotton cotyledon and embryo axis were extracted according to a modified Bligh and Dyer method [55]. Briefly, 300 µL MeOH were added to a known weight of frozen-dried cotton tissues was homogenised with metal bead on TissueLyser II (Qiagen) for 5 min at frequency of 30 Hz. Samples were then extracted with 600 µL chloroform and 300 µL 0.1 M KCl, and re-extraction with 500 µL chloroform. The combined lipid extracts were evaporated under nitrogen, then resuspended in chloroform in concentration of approximately 100 µg/µL. Fatty acids methyl esters (FAMEs) were prepared from total lipid extract by adding 1 mL of 0.1 M Sodium Methoxide per mg total lipids and incubating at 90°C for 1 h. The FAMEs were extracted with 1 mL H_2_O and 1 mL Hexane, and evaporated under nitrogen and resuspended in hexane. The analysis of FAMEs was performed on an Agilent 6890N gas chromatograph with a 30 m BPX70 column as previously described [56].

### Gossypol Analysis by HPLC

The embryo axis and cotyledon were separately dissected out from a mature seed and mid-maturity developing seed from *G. hirsutum* cv. Coker315. Following frozen-dry in liquid nitrogen and lyophilisation for 24 h, each sample was ground to a fine powder with a mortar and pestle and approximately 100 mg was used for gossypol analysis. Following extraction in 10 mL of 80% acetonitrile by sonication for 3 min, the samples were centrifuged at 2,800×g for 5 min prior to transfer of supernatant to a fresh tube. Samples were loaded onto a Waters ILC-2 Ion/Liquid Chromatography equipped with a diode array detector and autoinjector (WISP 710B), and eluted isocratically from a 150×3.9 mm i.d. Waters C18 Novapak column at 40°C, at 1 mL min^−1^ for a run time of 7 min, and the signal was monitored at 254 nm. Gossypol acetic acid (Sigma-Aldrich, St. Louis, MO, USA) was dissolved to a 0.01% solution in extraction solvent for generation of a standard curve for gossypol. Data collection and integration was carried out using Waters 840 Data and Chromatography Control Station software version 4.0.

## Supporting Information

Figure S1 **Real time qRT-PCR validation for selected unigenes.**
(DOCX)

Table S1 **A list of miRNAs in cotton developing embryos.**
(XLSX)

Table S2 **A list of genes encoding transcription factors.**
(XLSX)

Table S3 **A list of genes involved in providing pyruvate for fatty acid synthesis.**
(XLSX)

Table S4 **A list of galactosyltransferases involved in oligosaccharides synthesis.**
(XLSX)

Table S5 **Expression profile of genes involved in lipid metabolism.**
(XLSX)

Table S6 **Expression profile of seed storage proteins, LEA and LTP genes.**
(XLSX)

Table S7 **A list of genes involved in gossypol biosynthesis.**
(XLSX)

Table S8 **A list of primer sequences used for real time qRT-PCR.**
(XLSX)
