# Supplementary material for: Comparative Transcriptomic Analysis of Developing Cotton Cotyledons and Embryo Axis
Source: PLoS One. 2013 Aug 20;8(8):e71756. doi: 10.1371/journal.pone.0071756 (PMC3748104; doi:10.1371/journal.pone.0071756)
Supplement: Supplementary file 1 [file pone.0071756.s001.docx]

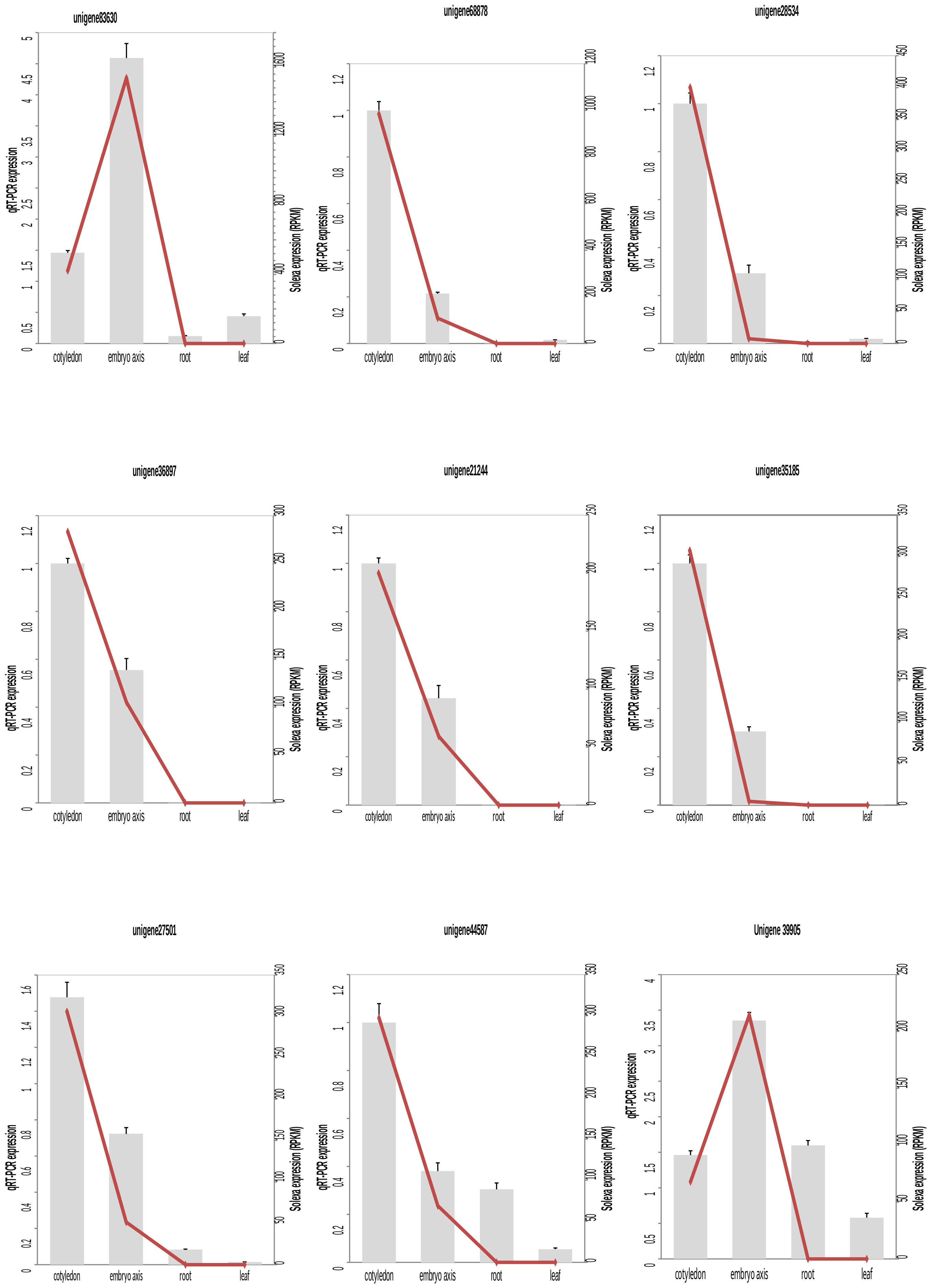

unigene83630
unigene68878
unigene28534
unigene36897
unigene21244
unigene35185
unigene27501
unigene44587
Unigene 39905
qRT-PCR expression
Solexa expression (RPKM)
cotyledon
embryo axis
root
leaf

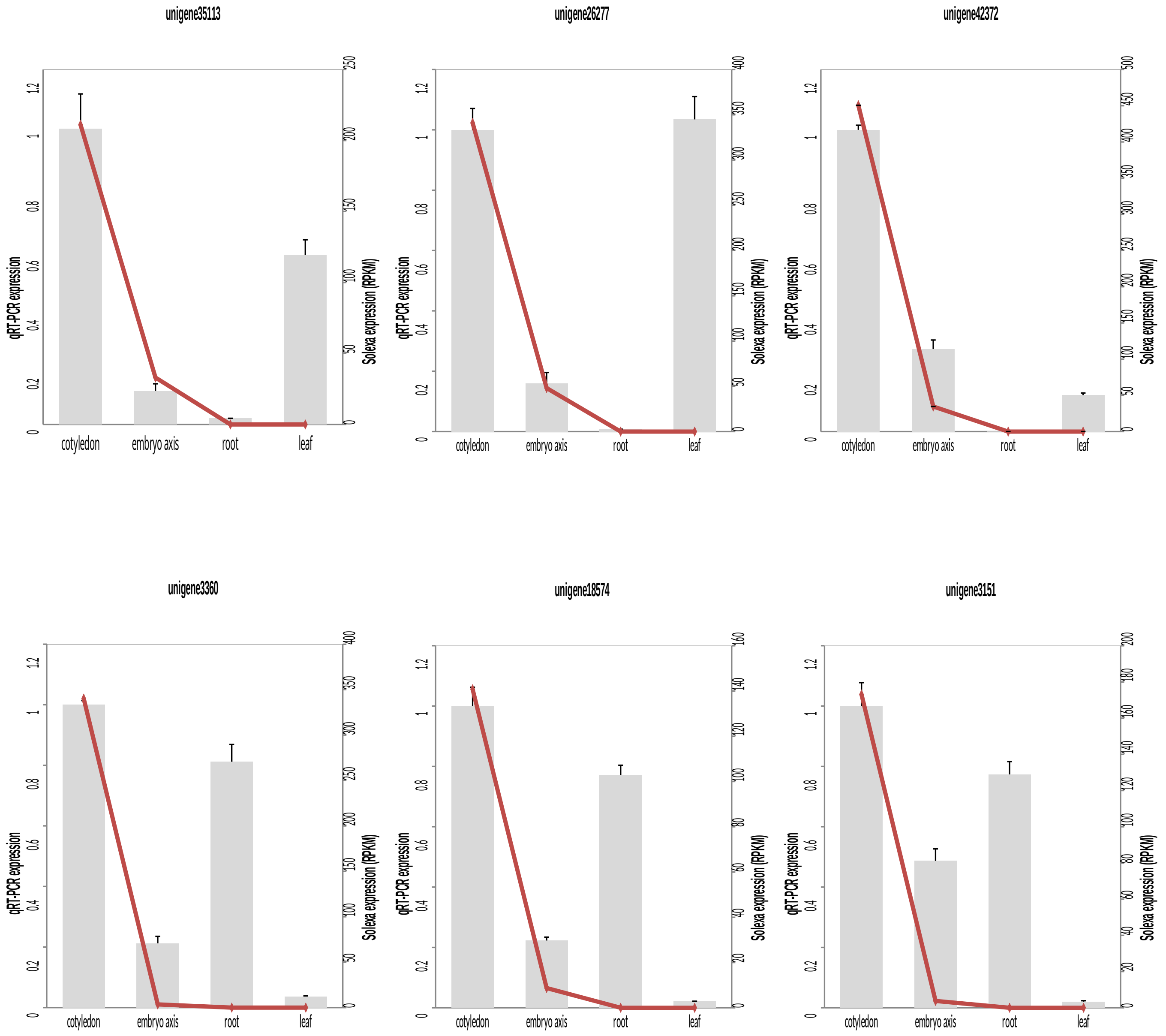
unigene35113
unigene26277
unigene42372
unigene3360
unigene18574
unigene3151
qRT-PCR expression
Solexa expression (RPKM)
0
0.2
0.4
0.6
0.8
1
1.2
cotyledon
embryo axis
root
leaf
0
50
100
150
200
250
300
350
400
450
500
20
40
60
80
120
140
160
180

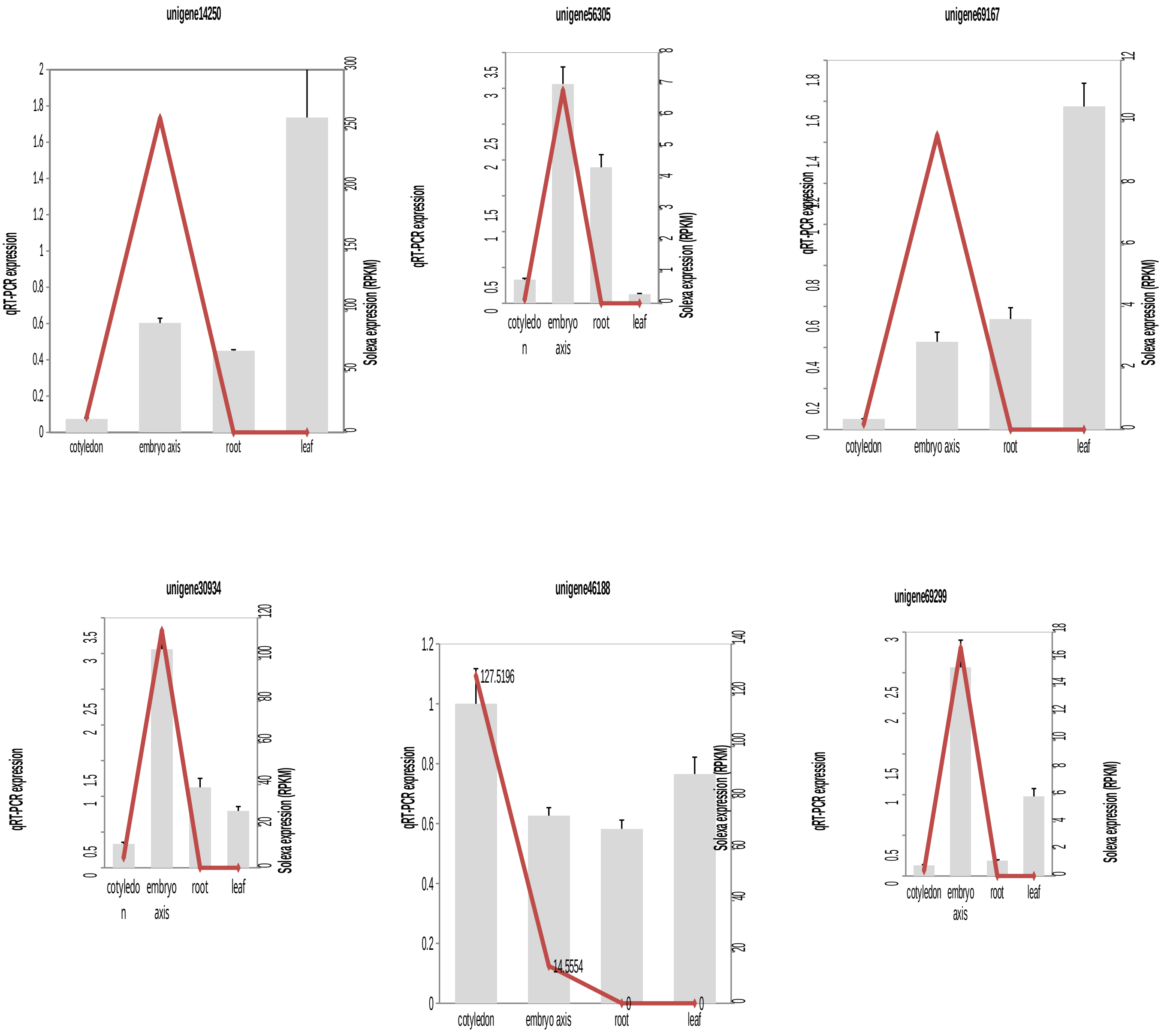
unigene14250
qRT-PCR expression
Solexa expression (RPKM)
cotyledon
embryo axis
root
leaf
unigene56305
qRT-PCR expression
Solexa expression (RPKM)
cotyledo n
embryo axis
root
leaf
unigene69167
qRT-PCR expression
Solexa expression (RPKM)
cotyledon
embryo axis
root
leaf
unigene30934
qRT-PCR expression
Solexa expression (RPKM)
cotyledo n
embryo axis
root
leaf
unigene46188
qRT-PCR expression
Solexa expression (RPKM)
127.5196
14.5554
0
0
cotyledon
embryo axis
root
leaf
unigene69299
qRT-PCR expression
Solexa expression (RPKM)
cotyledon
embryo axis
root
leaf
